# The relationship between deiodinase activity and inflammatory responses under the stimulation of uremic toxins

**DOI:** 10.1186/s12967-014-0239-5

**Published:** 2014-08-31

**Authors:** Gaosi Xu, Weiping Tu, Shulan Qin

**Affiliations:** Nanchang University, Nanchang, China; Department of Nephrology, Second Affiliated Hospital, Nanchang University, 330006 Nanchang, China; Department of Endocrinology, Third Affiliated Hospital, Nanchang University, 330008 Nanchang, China

**Keywords:** Uremic toxins, Small interfering ribonucleic acid, Inflammatory cytokines, Deiodinase

## Abstract

**Background:**

It is unclear to what extent uremic toxins participate in inflammatory responses and the activities of deiodinases, as well as the effects of deiodinases on inflammatory cytokines.

**Materials and methods:**

Hepatocellular carcinoma cell lines (HepG2) were transfected with small interfering ribonucleic acid (siRNA) specific for deiodinase type 1 (DIO1) and cultured with or without uremic toxins. The mRNA expression of DIO1, interleukin (IL)-1β, IL-6, and tumor necrosis factor (TNF)-α was detected by quantitative real-time PCR. The presence of selenoprotein M (SelM) and DIO1 was assessed by western blotting. Sonicate deiodinase activities in HepG2 cells were measured by a dithiothreitol-stimulated assay. The NF-κB, AP-1 and CREB-1 inflammatory signal pathways were confirmed by EMSA.

**Results:**

After culturing for 24 h, the mRNA expression of DIO1 was significantly decreased by the specific siRNA (reduced by 76%, *P* = 0.0002). Uremic toxins significantly increased the mRNA expression (*P* < 0.01) of IL-1β, IL-6 and TNF-α and inhibited DIO1 mRNA expression (*P* < 0.01) compared with controls. Suppression of DIO1 by siRNA significantly decreased the mRNA expression of IL-1β and IL-6 (*P* < 0.05) but not TNF-α (*P* = 0.093). Uremic toxins and specific siRNA synchronously reduced the protein expression of SelM and DIO1.

**Conclusions:**

Uremic toxins activate the expression of inflammatory cytokines. The major findings of this study indicate that the uremic toxins, more than inflammatory cytokines, play direct inhibitory roles in DIO1 enzyme activity, which then provides a negative feedback to the growing accumulation of inflammatory cytokines.

## Introduction

Nonthyroidal illness syndrome (NTIS), also known as euthyroid sick syndrome, refers to the characteristic changes in thyroid hormone levels, typically decreases in serum 3,5,3’-triiodothyronine (T3) levels and a reduction in the T3/reverse T3 (rT3) ratio, in critically ill patients. However, the pathogenesis of these imbalances in endocrine hormones is not fully understood [1]. The iodothyronine deiodinases (DIOs) modify the bioactivity of thyroid hormone by controlling the concentrations of thyroxine (T4) and its active form, T3. In humans, 80-90% of circulating T3 is derived from the pro-hormone T4, and the conversion from T4 to T3 is catalyzed by both type 2 deiodinase (DIO2) and, primarily, by type 1 (DIO1) via outer-ring deiodination [2]. The third deiodinase, DIO3, prevents T4 activation and terminates T3 actions. DIO1 is susceptible to inhibitory drugs, as well as changes in the thyroid hormone status. The prompt reduction of circulating T3 levels observed in NTIS must be, at least in part, due to the decreased peripheral conversion by DIO1 and/or DIO2 [3].

Available data suggest that interleukin (IL)-1β, IL-6, and tumor necrosis factor (TNF)-α play roles in the pathogenesis of NTIS. Serum IL-6 concentrations were negatively correlated with free T3 (FT3) and positively with rT3 concentrations [4]. Serum IL-6 is often increased in NTIS, and the serum level of this cytokine is inversely correlated with that of T3 in patients with end stage renal disease (ESRD) [5]. A single intravenous injection of IL-6 to healthy humans results in a transient decline in serum T3 and an increase in rT3; these changes mimic NTIS [6]. DIO1 is positively regulated by T3 and expressed in liver, kidney, thyroid, and pituitary. However, studies in mouse hepatic cells have shown that TNF-α, IL-1β, and IL-6 induce increases in DIO1 activity [7]. The role of DIO1 in NTIS is also being challenged because of the publication of data in which DIO1-deficient mice maintained normal T3 levels, which suggests that the decrease in liver DIO1 could be a consequence rather than a cause of reduced T3 [8]. Therefore, a better understanding of the mechanisms underlying altered DIO activities during uremic toxins exposure and illness is necessary to understand NTIS and ultimately improve the disease outcome in patients with chronic kidney disease.

Selenium (Se), which assists cells in resisting oxidative stress, is an essential trace element for mammals and may function as a redox regulator. Up-expression of selenoprotein M (SelM) in rats enhanced the activity of antioxidant enzymes such as glutathione peroxidase (GSH-Px) and superoxide dismutase (SOD) [9]. Moreover, SelM knockdown resulted in increased reactive oxidative stress (ROS), which further demonstrated the functional importance of SelM in preventing oxidative stress [10]. The addition of the antioxidant N-acetyl-cysteine (NAC), which restores intracellular GSH levels, prevented the IL-6-induced inhibitory effects on T3-mediated production, suggesting that the effect of IL-6 on DIO1 is mediated by oxidative stress [11]. Although little is known about the role of DIO2 in NTIS, all three deiodinases requires an endogenous cofactor, most likely GSH, which acts as a reducing agent to release iodine from the selenocysteine residue and regenerate the enzyme. However, little is known about the relationship between deiodinase and SelM during oxidative stress and inflammation.

DIO1 is susceptible to inhibitory drugs. It is unclear to what extent the uremic toxins play roles in the inflammatory responses and deiodinase activities in patients with chronic renal failure. In this study, by mimicking the internal environment of patients with chronic renal failure, we examine the effects of small interfering RNAs (siRNA) and uremic toxins on the expression and function of DIO1 and inflammatory cytokines. In this way, we are able to unveil the interaction among inflammatory cytokines, the activities of DIO1 and their possible signaling pathways during treatment with uremic toxins.

## Materials and methods

### HepG2 cell culture

HepG2 cells (human hepatoma) obtained from the Chinese Academy of Medical Sciences were grown in Dulbecco’s modified Eagle’s medium (DMEM) + 10% fetal bovine serum (FBS, v/v) + 100 nmol/l Se containing 100 units/ml penicillin and 30 μg/ml streptomycin under 5% CO_2_.

### RNA interference of DIO1

For the transfection of siRNAs, HepG2 cells were cultured and then transfected at a cell confluency of approximately 50% with Lipofectamine 2000, as indicated by the manufacturer’s instructions. Six hours after transfection, cells were collected and processed for quantitative real-time PCR (qRT-PCR) and fluorescence microscopy to evaluate the efficiency of siRNA knockdown. The siRNAs for DIO1 were chemically synthesized by Sangon Biotech, Shanghai, China. The siRNA sequences used were selected as follows: DIO1 sequence 1 (Seq1), 5’-GCCACUCUGAUACCAAGUATT-3’ and 5’-UACUUGGUAUCAGAGUGGCTT-3’; Seq2, 5’-GCUCUCUGUACCCUGAAAUTT-3’ and 5’-AUUUCAGGGUACAGAGAGCTT-3’; Seq3, 5’-GGCUGUGACUUGAUUCAAATT-3’ and 5’-UUUGAAUCAAGUCACAGCCTT-3’. Sequence 5’-UUCUCCGAACGUGUCACGUTT-3’ and 5’-ACGUGACACGUUCGGAGAATT-3’ for negative control FAM. Sequence for negative control, 5’-UUCUCCGAACGUGUCACGUTT-3’ and 5’-ACGUGACACGUUCGGAGAATT-3’. 5’-GUAUGACAACAGCCUCAAGTT-3’ and 5’-CUUGAGGCUGUUGUCAUACTT-3’ for GAPGH positive control. After evaluation by qRT-PCR and fluorescence microscopy, Seq3 was selected as the final siRNA because of its optimal interference efficiency above 76%.

### Study groups

The study groups were configured as following: Controls, DMEM + 10% FBS + 100 nmol/l Se; Toxins, controls + 80 μmol/l urea + 200 μmol/l creatinine + 10 nmol/l uric acid + 2 pg/ml parathyrin and 20 μmol/l spermidine (final concentrations); SiRNAs, controls + siRNAs; and the combined toxins and siRNAs group. All cells were cultured and collected for analysis at 3 h, 6 h, 12 h and 24 h.

### RNA extraction and qRT-PCR

Total RNA was isolated from HepG2 cells using an RNeasy mini kit (Qiagen, Texas, USA) according to the manufacturer’s specifications. Total RNA from each sample was reverse-transcribed with random primers using a StrataScript reverse transcriptase kit (Stratagene, California, USA) followed by qRT-PCR analysis. The primers for DIO1 amplification were 5’-TGGTGGTGATGATGGGTGAG-3’ and 5’-GCATTGCTGGTGCCTATTGTT-3’. The primers for IL-1β were 5’-ACGAATCTCCGACCACCACTA-3’ and 5’-GCACATAAGCCTCGTTATCCC-3’. IL-6 were 5’-TGAGGAGACTTGCCTGGTGAA-3’ and 5’-GCATTTGTGGTTGGGTCAGG-3’. The primers for TNF-α gene amplification were 5’- GGCAGTCAGATCATCTTCTCGAA-3’ and 5’- TGAAGAGGACCTGGGAGTAGATG-3’. The primers for house-keeping gene GAPDH amplification were 5’-GGAGTCCACTGGCGTCTTC-3’ and 5’-GCTGATGATCTTGAGGCTGTTG-3’. The qRT-PCR was performed with Taqman One-step RT-PCR Master Mix Reagents on an ABI Prism 7700 Sequence Detection System (Applied Biosystems, Foster city, CA). The singularity and specificity of amplifications were evaluated by Dissociation Analysis Software (ABI PRISM Com., CA, USA). All reactions were performed in triplicate. Relative expression levels and standard deviations were calculated using the comparative method.

### DIO1 activity

To determine the activity of DIO1, HepG2 cells were prepared and assayed, as previously described [12]. Cells were washed with HBSS and homogenized in buffer containing 250 mmol/l glucose, 20 mmol/l Hepes, 1 mmol/l EDTA, and 1 mmol/l dithiothreitol by ultrasonication (ten pulses of 0.5 s at 200 W). The proteins were measured by a modified Bradford assay (Bio-Rad, Munich, Germany). Samples were then incubated in a total volume of 200 μl containing 1 μmol/l rT3, 50 000 cpm/tube [3’,5’-^125^I]rT3 (labeled using 17 Ci/mg carrier-free ^125^I from PerkinElmer, Massachusetts, USA), 0.05 mg microsomal protein/ml, 2 mmol/l EDTA, and 5 mmol/l dithiothreitol and incubated for 30 min at 37°C, followed by trichloroacetic acid (TCA) precipitation of media and counting of the ^125^I^−^ remaining in the supernatant. Non-specific cell deiodination was determined by the addition of 100 μmol/l propylthiouracil (PTU). All samples were measured in triplicate.

### Western blotting

Cells were harvested with ice-cold PBS and centrifuged at 14000 × g for 4 min at 4°C. Nuclear and cytosolic extracts were prepared using a Nuclear and Cytoplasmic Protein Extraction Kit (Pierce Biotechnology, Rockford, Illinois) according to the manufacturer’s instructions. Protein concentrations were measured by using a bicinchoninic acid protein assay kit (Pierce Biotechnology, Rockford, Illinois). Protein extractions of 50 μg were resolved in 10% SDS-PAGE and transferred onto immunoblot polyvinylidene difluoride membranes. Briefly, the blots were blocked with 5% nonfat milk, washed with Tris-buffered saline with 0.1% Tween (TBS-T), and incubated overnight at 4°C with primary rabbit antibodies DIO (1:400) (Santa Cruz Biotechnology, Dallas, Texas), SelM (1:1000) (Abcam, Cambridge, UK) and β-actin (the marker of cytoplasm fraction) (1:2000) (Santa Cruz Biotechnology). Blots were then washed in TBS-T and incubated with horseradish peroxidase-labeled secondary goat anti-rabbit (1:2000; Santa Cruz Biotechnology) for 1 h at room temperature. Western analysis of quantification was performed with Image J (National Institutes of Health/USA).

### Electrophoretic mobility shift assay (EMSA)

After culturing for 12 h, nuclear extracts were prepared using nuclei lysis solution, and the EMSA was performed with the NF-κB Gel Shift Assay System according to the manufacturer’s instructions. Protein concentrations were determined using the BioRad protein reagents. The NF-κB double-stranded consensus oligonucleotide 5’-AGTTGAGGGGACTTTCCCAGGC-3’ was end-labeled with Cy5.5-lectin. Unincorporated nucleotides were removed by passing the reaction mixture through a Sephadex G-25 spin column (Amersham-Pharmacia, Uppsala, Sweden). Purified Cy5.5-lectin-labeled probe was incubated in 25 μl total volume binding reaction mixture containing 5 μg nuclear extracts at room temperature for 30 min. DNA-protein complexes were separated by electrophoresis through a 6% non-denaturing acrylamide:bis-acrylamide (37.5:1) gel in 0.5 × Tris-borate/EDTA (TBE) for 2 h at 150 V. The NF-κB 5’-GCCTGGGAAAGTCCCCTCAACT-3’ unlabeled double-stranded consensus oligonucleotide was included in 2-fold molar excess over the amount of Cy5.5-lectin-labeled NF-κB probe to detect specific and non-specific DNA-protein interactions. The composition of the complexes was determined by supershift assays. Quantification was performed with Image J (National Institutes of Health/USA). The double-stranded consensus oligonucleotide was 5’-CGCTTGATGACTCAGCCGGAA-3’ for the acute protein (AP)-1 assay, and the unlabeled double-stranded consensus oligonucleotide was 5’-TTCCGGCTGAGTTACCAAGCG-3’ for its competition. Sequence 5’-AGAGATTGCCTGACGTCAGAGAGCTAG-3’ and unlabeled 5’-CTAGCTCTCTGACGTCAGGCAATCTCT-3’ were used for the cAMP response element-binding protein (CREB)-1.

### Measurement of IL-6 in culture supernatants by ELISA

IL-6 production was measured in culture supernatants by using a Human IL-6 Quantikine ELISA kit (R&D systems, Minneapolis, MN) according to the manufacturer’s instructions. All samples were measured in triplicate.

### Statistical analysis

Raw mRNA expression data were computed by 2^−^△^Ct^ formula with values normalized to GAPDH [13]. All data were presented as the mean ± standard deviation (SD) of triplicate experiments, unless otherwise specified. Data were analyzed by using Student’s 2-tailed T test or one-way ANOVA, followed by post-hoc Duncan multiple range test when *F* values were significant. SPSS software version 17.0 for Windows was used for statistical analysis. A *P* value less than 0.05 was considered significant.

## Results

### Estimating the mRNA expression by qRT-PCR

**Figure 1:**
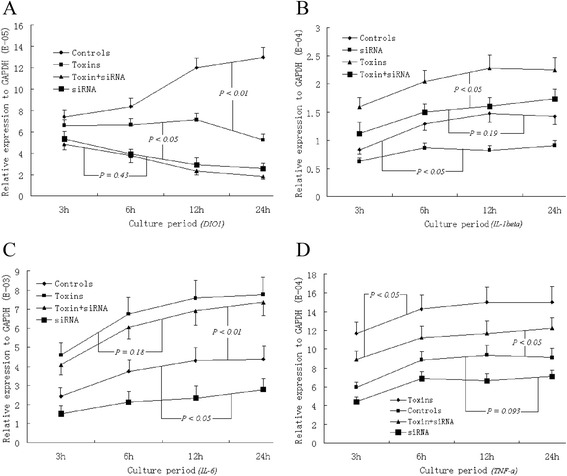
**Estimating the mRNA expression by quantitative real-time PCR of DIO1 (A), IL-1β (B), IL-6 (C), and TNF-α (D) in cultured HepG2 cells.** Samples were measured in triplicate, and data are shown as the mean ± standard deviation.

We evaluated the effects of uremic toxins and specific siRNA treatment on the temporary mRNA expression of DIO1 and inflammatory cytokines. The inhibition of DIO1 by specific siRNA treatment significantly decreased its mRNA expression compared with controls (*P* < 0.01) and uremic toxins (*P* < 0.05) (Figure 1A). Although the relative expression level was lower, 10^−5^ to GAPDH, uremic toxins markedly downregulated the DIO1 mRNA expression within the 24 h culture period. Uremic toxins significantly increased the mRNA expression of IL-1β (*P* < 0.05), IL-6 (*P* < 0.01) and TNF-α (*P* < 0.01) compared with controls (Figure 1B, C and D). Specific siRNA treatment for DIO1 notably decreased the mRNA levels of IL-1β (*P* < 0.05) and IL-6 (*P* < 0.05) but not TNF-α (*P* = 0.093). The quantitative values of IL-1β and TNF-α mRNA expression in HepG2 cells cultured with uremic toxins and specific siRNA were more prominently reduced (*P* < 0.05) than those cultured with only uremic toxins (*P* < 0.05), but that of IL-6 was not (*P* = 0.18). This result could be explained by the relatively higher mRNA expression compared with GAPDH (10^−3^); therefore, specific siRNA treatment may play a contrasting role to that of uremic toxins.

### DIO1 activities of HepG2 cells

**Figure 2:**
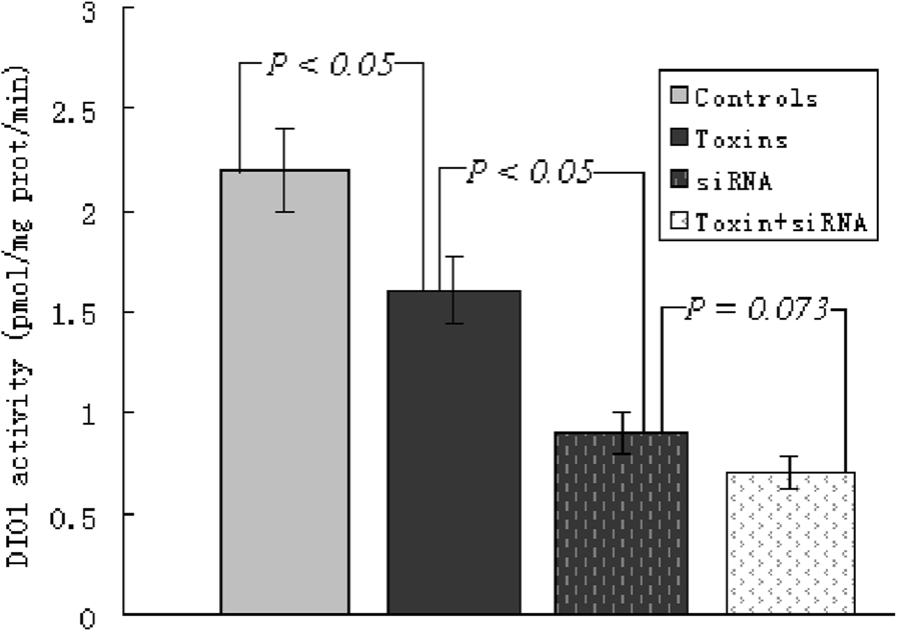
**Uremic toxins and/or siRNA induced decreases in DIO1 activities of HepG2 cells cultured for 12 h were paralleled by the declines in their respective expression of mRNA and protein (Figure** **3** **).** Samples were measured in triplicate, and data are shown as the mean ± standard deviation.

The deiodinase activities in the presence of dithiothreitol (DTT) were estimated for the maximal function of DIO1 in HepG2 cells. As indicated in Figure 2, although T3 production in the siRNA treatment did not differ from that in the case of treatment with siRNA and toxins (*P* = 0.073), significant decreases in T3 production were observed in the group treated with uremic toxins (the DIO1 activities decreased by nearly 27%, *P* < 0.05) and group siRNA (the DIO1 activities decreased by approximately 59%, *P* < 0.01) compared with controls. The decrease in DIO1-catalyzed T4-to-T3 conversion induced by siRNA and/or uremic toxins was measured at 12 h into incubation. Consistent with the results of qRT-PCR mRNA analysis, the specific siRNA treatments silenced the activities of the corresponding proteins.

### Western blotting analysis of DIO1 and SelM expression

**Figure 3:**
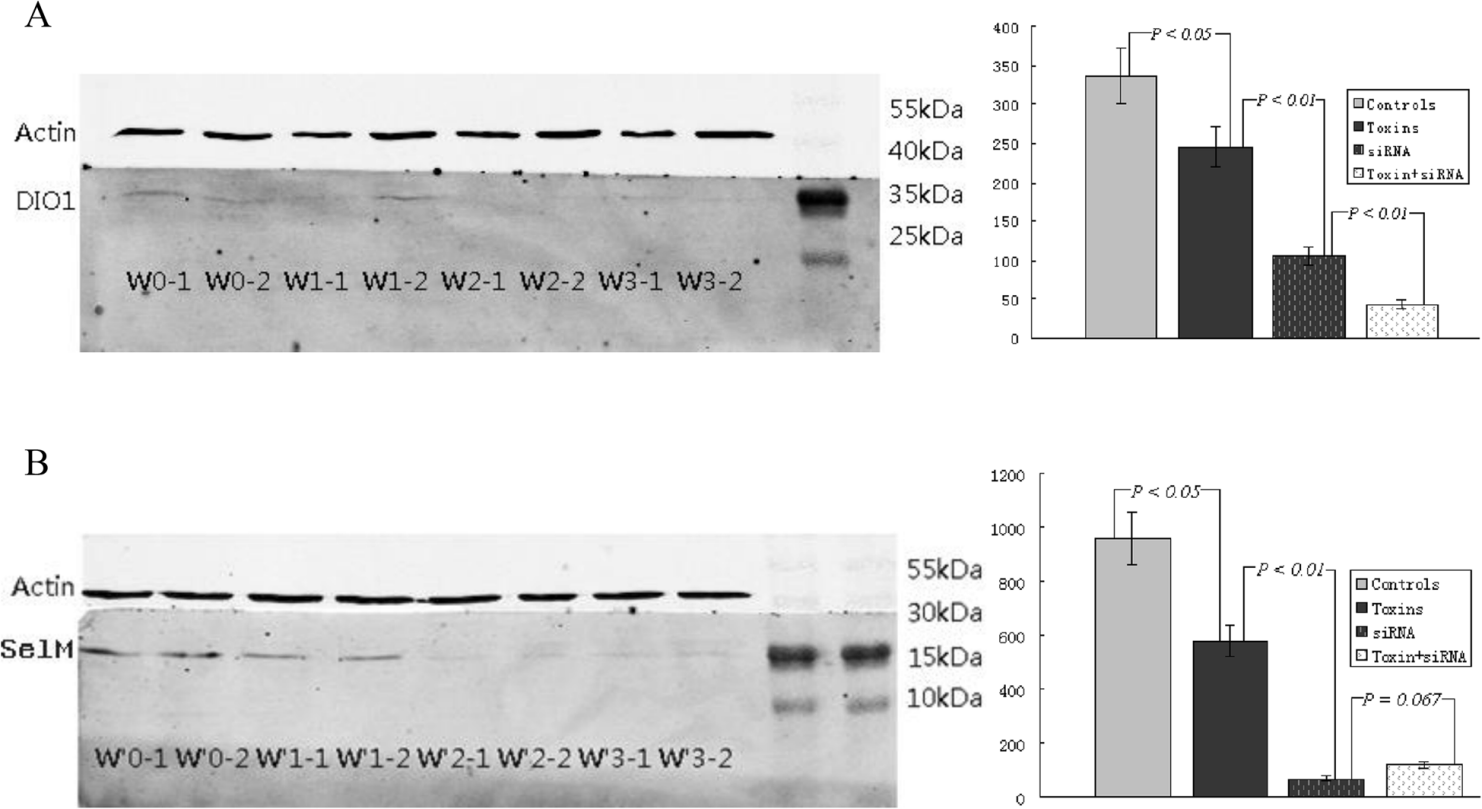
**Effects of uremic toxins and specific siRNA treatment on HepG2 cells cultured for 12 h (n = 4 in each group) by western blot.** Normalized densitometric data of the DIO1 (28 KDa, **A**) and SelM (16.2 KDa, **B**) bands obtained from the protein extractions. W0 = controls, W1 = toxins, W2 = siRNA, and W3 = toxin + siRNA. Data are shown as the mean ± standard deviation.

At relatively low expression levels, the DIO1 protein (28 KDa) expression in HepG2 cells was significantly inhibited by approximately 28% (*P* < 0.05) after treatment with uremic toxins and approximately 58% (*P* < 0.01) after treatment with the specific siRNA (Figure 3A), compared with controls. Because all 3 deiodinases and SelM are selenocysteine-containing, thiol-interacting oxidoreductases, these enzymes were speculated to be similarly sensitive to the redox changes. Therefore, we also evaluated SelM protein expression by immunoblotting. SelM protein (16.2 KDa) expression was significantly inhibited by approximately 40% (*P* < 0.05) after treatment with uremic toxins and approximately 92% (*P* < 0.01) after treatments with the specific siRNA (Figure 3B), compared with controls.

Confocal microscopy studies have indicated that DIO1 is localized in the plasma membrane, with its active center predicted to be in the cytosol. IL-6 can alter GSH/oxidized GSH shuttling and recycling, which leads to intracellular GSH depletion as a result of ROS formation [14]. Because the decreased SelM protein expression induced by uremic toxins and/or siRNA was paralleled by the DIO1 activities, which was also confirmed by western blot, we speculated that the reduction in DIO1 function in HepG2 cells could be due to the inhibition of GSH by the generation of ROS and subsequent oxidative stress. Therefore, we deduced that, except for the inhibitive effects on DIO1 mRNA expression and enzyme activities by uremic toxins, the subsequently produced inflammatory cytokines, especially IL-6, may also play important roles in the suppression of DIO1.

### Inflammatory signal pathways by EMSA

**Figure 4:**
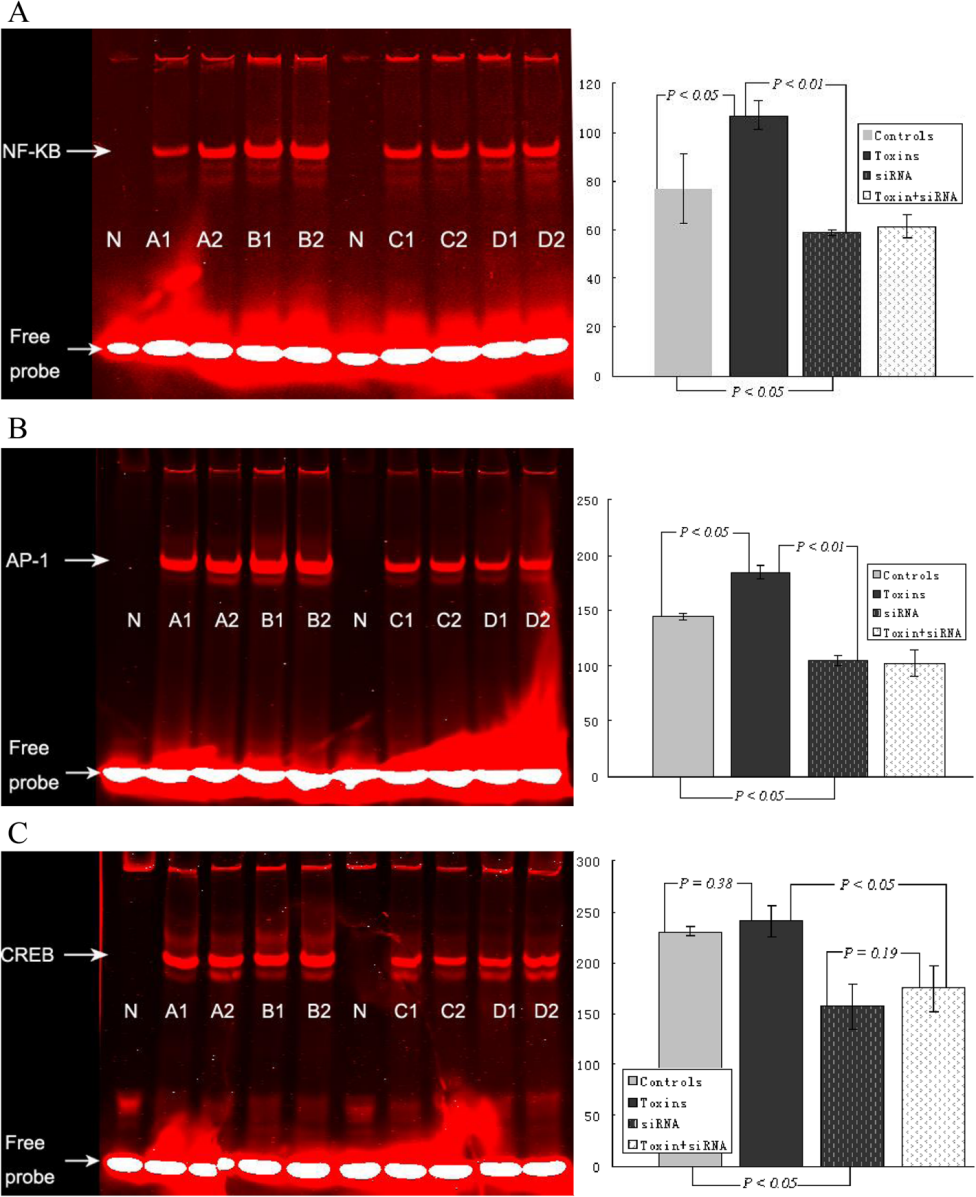
**Effects of uremic toxins and specific siRNA treatment on NF-κB (A), AP-1 (B) and CREB-1 (C) activation by EMSA in HepG2 cells cultured for 12 h (n = 4 in each group).** Normalized densitometric data of the NF-κB, AP-1, and CREB-1 bands obtained from the extracts of proteins with unclear interactions. N = negative control, **A** = controls, **B** = toxins, **C** = siRNA, and **D** = toxin + siRNA. Data are shown as the mean ± standard deviation.

For DNA binding activities that were unclear, extracts were evaluated by EMSA, and we observed that the uremic toxins and/or specific siRNA treatments inactivated the inflammatory signals involving NF-κB, AP-1 and CREB-1 pathways. In contrast to controls, there was a prominent activation of NF-κB in HepG2 cells cultured with uremic toxins (*P* < 0.05) and inhibition in cells with specific siRNA treatment (*P* < 0.05, Figure 4A). The NF-κB/DNA complex was displaced by an excess of unlabeled NF-κB, and not by Cy5.5-lectin-labeled NF-κB probe, demonstrating the specificity of the NF-κB/DNA binding complex.

Inflammatory cytokines such as IL-1 and TNF-α were not only regulated by NF-κВ but also by activator protein AP-1 [15]. Additionally, CREB is a known inducer of DIO1, DIO2 and DIO3 gene transcription [16,17]. Therefore, we speculated that AP-1 and CREB pathways are also involved in the inflammatory responses. Compared with controls, specific siRNA treatment reduced AP-1 signals by approximately 28% (*P* < 0.05, Figure 4B) and approximately 32% for CREB-1 (*P* < 0.05, Figure 4C). Interestingly, paralleled by the mRNA expression of inflammatory cytokines and confirmed by qRT-PCR, uremic toxins significantly increased the production of AP-1 protein (*P* < 0.05), but not CREB-1 (*P* = 0.38).

### ELISA screening for IL-6

**Figure 5:**
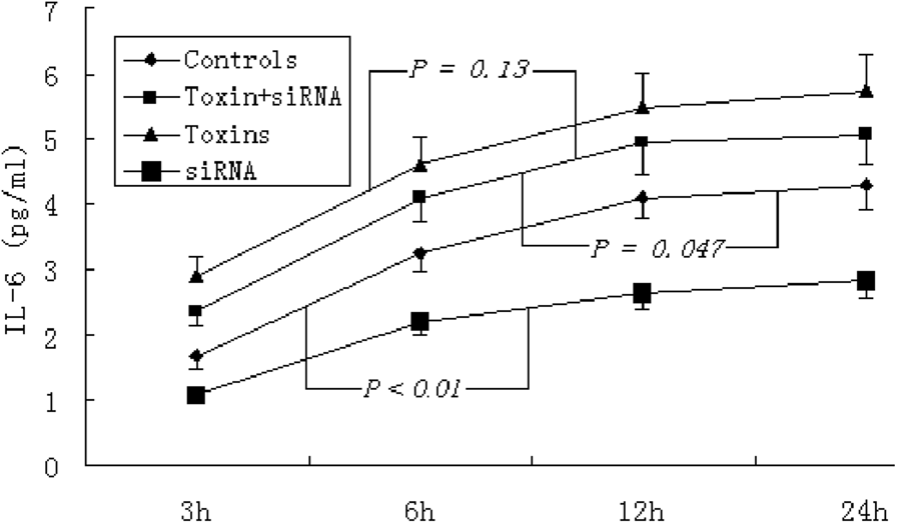
**ELISA quantification of IL-6 concentrations in culture supernatants of HepG2 cells.** Data are shown as mean values, and all samples were measured in triplicate.

Concentrations of IL-6 in culture supernatants, measured by ELISA, increased time-dependently during the 12 h culturing period. In contrast to the stimulated secretion of IL-6 due to uremic toxins (*P* < 0.05 versus controls), specific DIO1 siRNA treatment (*P* < 0.01 versus controls) depressed the production of IL-6 in HepG2 cells (Figure 5). Interestingly, there is no distinct difference in IL-6 production between cells cultured with uremic toxins and those cultured with uremic toxins and specific siRNA (*P* = 0.13). This phenomenon was observed previously in the quantitative analysis of IL-6 mRNA expression, which has been interpreted by the limited siRNA effects in the face of eminent activation by uremic toxins. The stimulation of IL-6 by uremic toxins and the inhibitory effects induced by specific siRNA were consistent with the results from EMSA, which further confirmed that the uremic toxins play pivotal roles in the inflammatory responses of patients with chronic renal failure.

## Discussion

IL-6 decreases hepatocyte thyroxine deiodinase expression and inhibits thyroid function through the binding of IL-6 and soluble IL-6 receptor (sIL-6R) complex to gp130 [18]. The infusion of recombinant TNF-α in human also produced a significant decrease in serum T3 [19]. Cytokines inhibit the mRNA expression and function of DIO1 in HepG2 cells, and studies of rat hepatocyte cells has indicated that IL-1 and IL-6 impair T3-mediated induction of DIO1 expression by a mechanism involving thyroid hormone receptor interaction [20,21]. Blocking with IL-6, however, failed to induce the euthyroid sick syndrome (also called NTIS) in animal models [22]. In an experimental IL-6 knock-out mouse study, a less distinct decrease in T3 levels and alternation of liver D1 activity were found after administration of lipopolysaccharide (LPS), which prompted questions regarding the clinical findings [23].

Therefore, it is necessary to observe the exact mechanisms of interaction between inflammatory cytokines and DIOs from a new perspective in which we discuss the relationship inversely from DIOs to cytokines by gene silencing of deiodinase. Moreover, the effects of uremic toxins *per se* on inflammatory responses and DIO activities in critical illness have not been previously investigated. Confirmed by qRT-PCR, DTT estimation of DIO1 activities, western blot, EMSA and ELISA assays, we concluded that the specific siRNA treatment not only decreased the DIO1 mRNA expression and enzyme activities but also played an inhibitory role in the production of inflammatory cytokines in cultured HepG2 cells. The major finding of the present study is that the uremic toxins *per se*, more than inflammatory cytokines, play a pivotal role in the inhibition of DIO1 activity.

Oxidative stress due to augmented ROS or reactive nitrogen species generation is characteristic of critical diseases associated with NTIS. The patients usually display decreased plasma and intracellular levels of antioxidant scavenging molecules, such as GSH, as well as reduced activity of the antioxidant enzymatic system involving ROS detoxification [24]. By increasing intracellular GSH concentrations, NAC completely diminished the inhibitory effect of IL-6 on D1 and D2-mediated T4 to T3 conversion [11]. The effects of selenium deficiency on hepatic and thyroidal DIO1 and selenium-dependent glutathione peroxidase (GSH-Px) activities have been studied in weanling rats. In selenium-deficient rats, hepatic DIO1 activity was reduced to 11%. Their hepatic and GSH-Px activities were also reduced [25]. Confirmed by qRT-PCR, DTT estimation of DIO1 activities and western blot analyses, uremic toxins and specific siRNA synchronously reduced the protein expression of SelM and DIO1, which further verified the role of inflammatory cytokines in decreasing deiodinase activities.

NF-κВ activation is a major pathway involved in the process of inflammation where IL-1 and TNF-α is pivotal players. Activation of NF-κВ promotes the release of inflammatory mediators including IL-1, IL-6 and TNF-α [26]. TNF-α activates NF-κB pathways, and then inhibits DIO1 mRNA and enzyme activity in HepG2 cells [27]. Inflammatory cytokines such as IL-1 and TNF-α were not only regulated by NF-κВ but also by both activator protein AP-1 [15] and CREB, a known inducer of DIOs gene transcription [17]. The present EMSA results suggest that uremic toxins promoted the NF-κВ, AP-1 and CREB protein expression, most likely by increasing the production of inflammatory cytokines.

**Figure 6:**
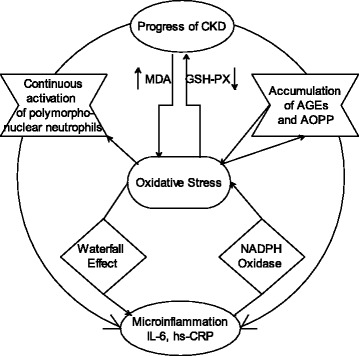
**The simplified possible pathogenesis between inflammation and oxidative stress in patients with chronic kidney disease (CKD).** AGEs: advanced glycation end products, AOPP: advanced oxidation protein products, NADPH: nicotinamide adenine dinucleotide phosphate.

Although the exact mechanisms of inflammation and oxidative stress have not been accurately elucidated in CKD patients, a number of the following factors appear to be involved, including uremic toxins [28], rennin-angiotensin system [29], hypertension [30], underlying diseases (diabetes and autoimmune diseases, etc.) [31], infection, iron overload [32], and antioxidant deficiency [33], etc. Oxidative stress can provoke inflammation by activating NF-κB and causing the subsequent generation of pro-inflammatory cytokines (Figure 6) [34]. The production and release of reactive oxygen or nitrogen species and chlorine by the activated immune cells, in turn, promotes oxidative stress [35]. However, the effects of uremic toxins *per se* on deiodinase activities and inflammatory responses, as well as the converse effect of deiodinases on inflammatory cytokines, have not been previously investigated.

**Figure 7:**
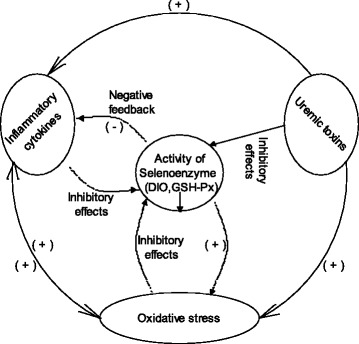
**Proposed mechanisms of the interaction between inflammatory cytokines, oxidative stress and deiodinase activities in patients with chronic renal failure.** (+): promoting effect, (-): inhibitory effect.

As indicated in Figure 7, inflammatory cytokines, uremic toxins, and oxidative stress play an inhibitory role in the activation of deiodinases. The present findings provide a possible mechanistic explanation for the decreased enzyme activities and increased inflammatory cytokines observed in the mimicked circumstances of chronic renal failure. The suppression of deiodinase activities conversely resulted in a strong inhibitory effect on the production of inflammatory mediators, providing negative feedback to avoid the cascading effect and to establish a new balance in the internal environment of patients with chronic renal failure.

There are several limitations in the present study. First, siRNA for DIO1 was transient, and vector-transfected siRNA should be investigated for a longer period in *in vivo* animal experiments. Second, there are three types of deiodinases, therefore, siRNA for DIO2 and/or DIO3 should be investigated. Third, the concentrations of uremic toxins in culture medium for HepG2 cells were an arbitrary simplex.

## Conclusions

The major findings of the present study are that the uremic toxins, more than inflammatory cytokines, play inhibitory roles in DIO1 enzyme activity, which then provides negative feedback to the growing concentrations of inflammatory cytokines.
